# The evolution of the *Journal* covers: 55 years of uninterrupted progress

**DOI:** 10.1051/ject/2023018

**Published:** 2023-06-28

**Authors:** Alfred H. Stammers

## A brief history

In the spring of 1961 a small group of individuals from diverse backgrounds all had one thing in common: They were performing a new technology called extracorporeal circulation (ECC). Less than a decade earlier surgeons and researchers had shown that patients suffering from cardiac disease could be placed on a ‘heart-lung’ machine for a short period of time facilitating intracardiac access for surgical repair. The first formal meeting for individuals practicing and studying ECC, using either ‘heart-lung’ or dialysis machines, was held in 1962. In 1963 the American Society of Extracorporeal Circulation Technicians was formed serving as the first professional society devoted to ECC, and included both perfusionists (a new professional group) and dialysis technicians, who were often nurses. The first formal meeting of AmSECT was held in Chicago at the Sol Fox Lounge in December 14, 1963. In 1967 the Society was formally incorporated with a name change to the American Society of Extra-Corporeal Technology,^1^ maintaining the familiar acronym AmSECT.

The following year, due to the undaunted efforts of perfusionists and surgeons, a new journal appeared devoted entirely to this emerging field, and was titled *The Journal of Extra-Corporeal Technology*, commonly referred to today as *JECT*. The goal was to publish the progress and growth of technological knowledge on ECC, and to share the research in a multidisciplinary manner. In the first issue of *JECT* Jim Wade, the president of AmSECT at the time, wrote in an editorial that the primary goal of both the Society and *JECT* would be the “communication and the professional exchange of information” by perfusionists, surgeons and administrators [1]. While individuals from numerous other professions have benefited from the 55-year history of the publication of *JECT*, the goals of the initial formation of the *Journal* remain similar. During those years there have been many changes to the *Journal* with perhaps the most obvious being the cover. This year the *Journal* will undergo its fifth major cover change so it may be fitting to review the history of the front page of this publication.

The first editor-in-chief of *JECT* was Ed Berger a practicing perfusionist from the Charles T. Miller Hospital in St. Paul, Minnesota. Ed was truly a visionary who saw the importance of the scientific pursuit of quality evidence as paramount in supporting methodologies and practices that would improve the conduct of ECC, with the ultimate goal of improving patient care. In his first editorial he established the focus of the *Journal* as a collaborative effort and stated “by us banding together for the acquiring and sharing of information in order to develop a more perfect technology and more perfect technologists” [2]. Indeed, today, as in 1968, these words remain the driving force for all of us as we commit obligation to our patients.

## The *Journal* covers

**Figure 1:**
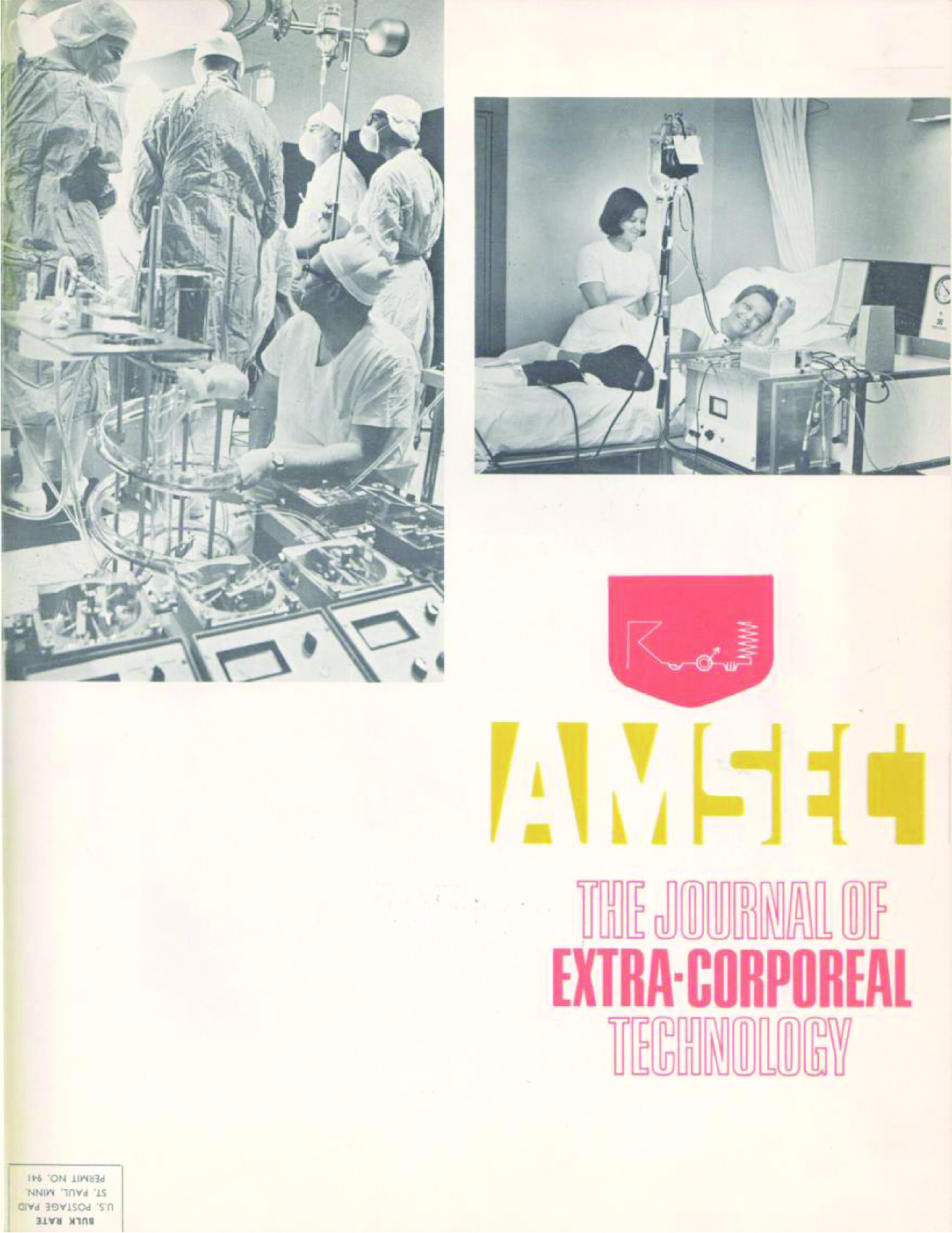
*The Journal of Extra-Corporeal Technology* cover. December 1968. Volume 1, Issue 1.

**Figure 2:**
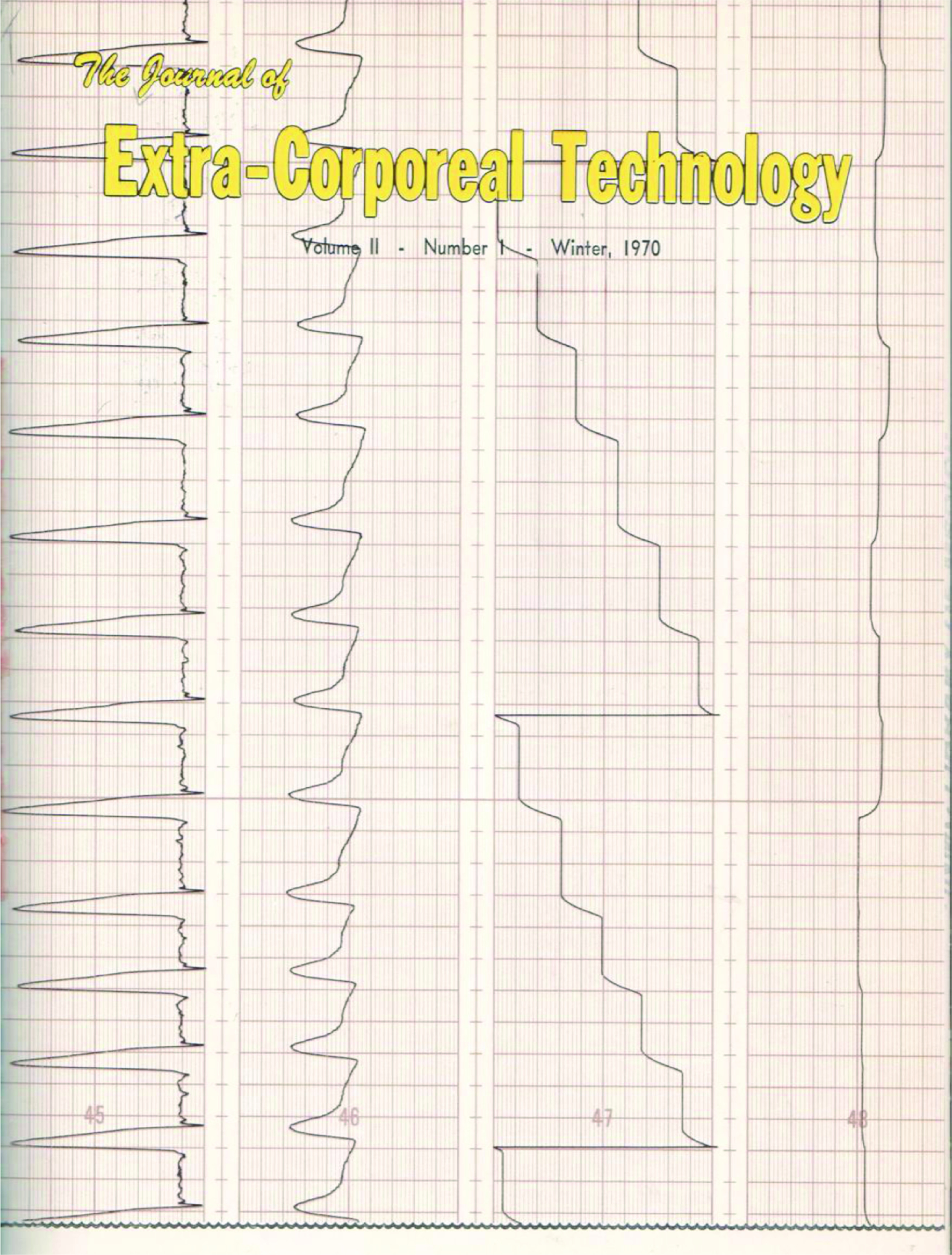
*The Journal of Extra-Corporeal Technology* cover. Winter 1970. Volume 2, Issue 1.

**Figure 3:**
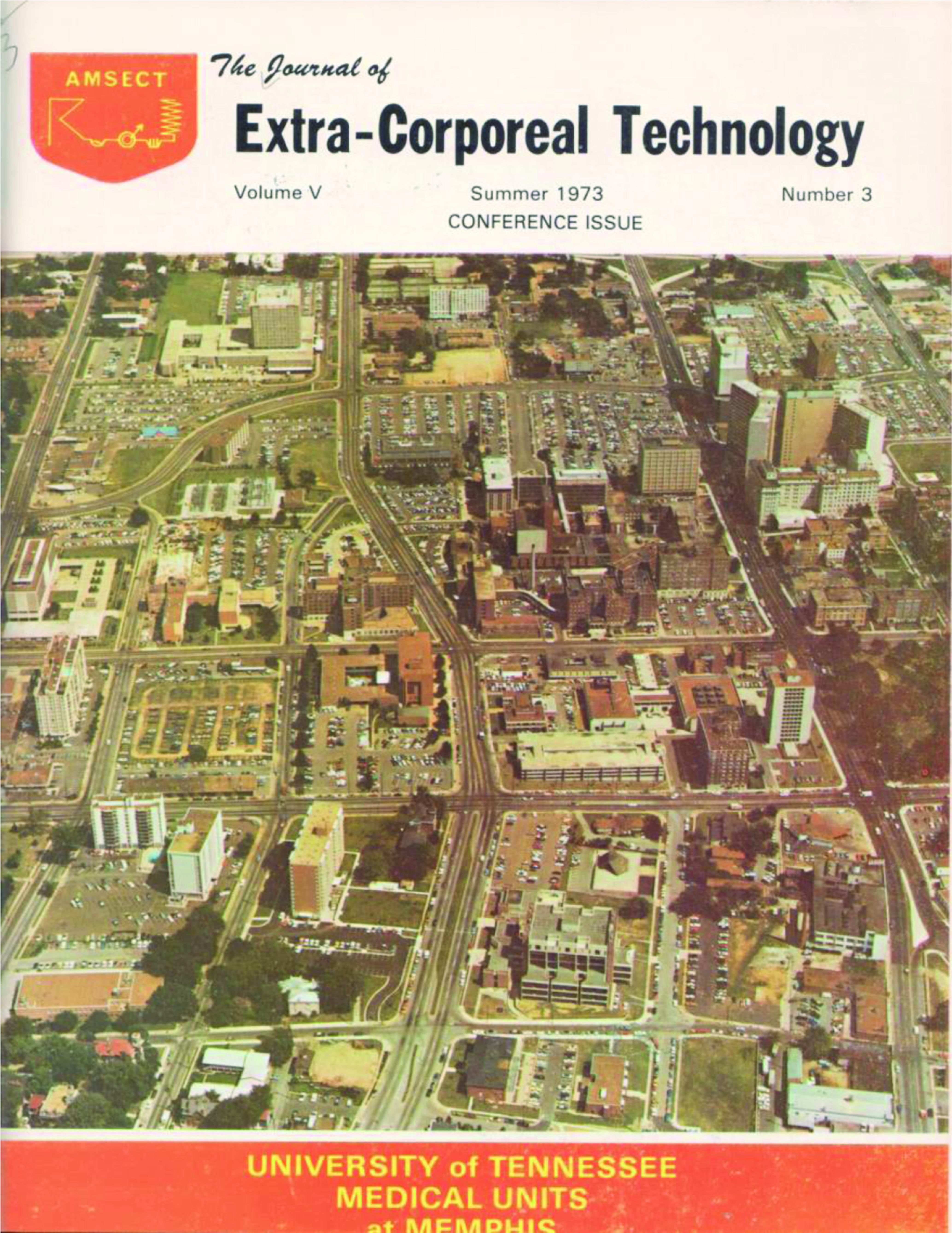
*The Journal of Extra-Corporeal Technology* cover. Summer 1973. Volume 5, Issue 3.

The first cover shows the early juxtaposition of perfusionists and dialysis technicians with images of both professions displayed (Figure 1). In fact the photo of the perfusionist using the DeWall-Lillehei bubble oxygenator appearing on the cover of Volume 1, Issue 1, published in December 1968, is Ed Berger. The dialysis machine seen on the right side of the image, is a Travenol RSP Hemodialysis System which used the twin-coil dialyzer designed by William Kolff.^2^ For the next eight years the cover changed with each issue and usually depicted a graphic that either reflected content found within the issue (Figure 2), or contained an image of a hospital where early AmSECT leaders worked (Figure 3).

**Figure 4:**
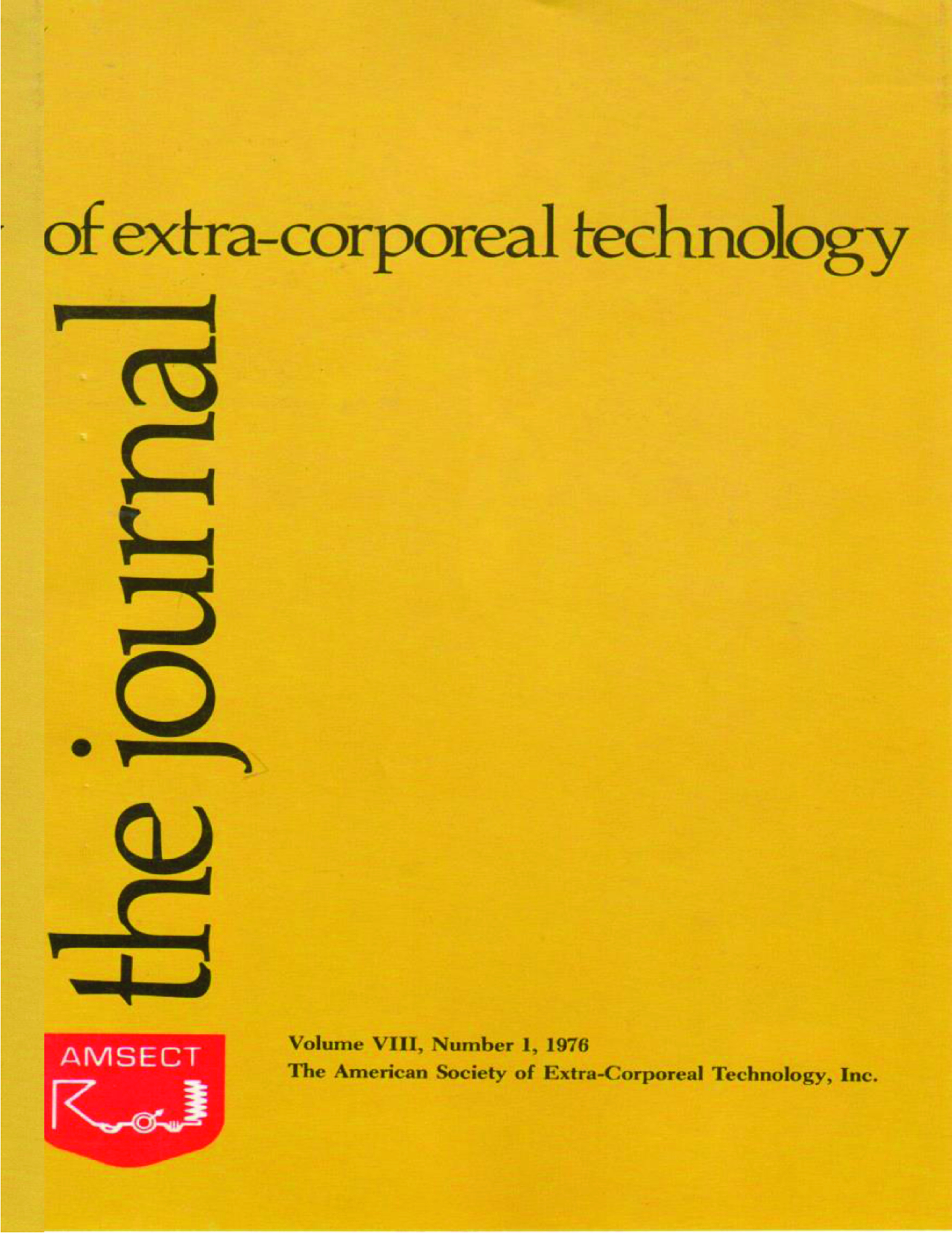
*The Journal of Extra-Corporeal Technology* cover. Summer 1976. Volume 8, Issue 1.

**Figure 5:**
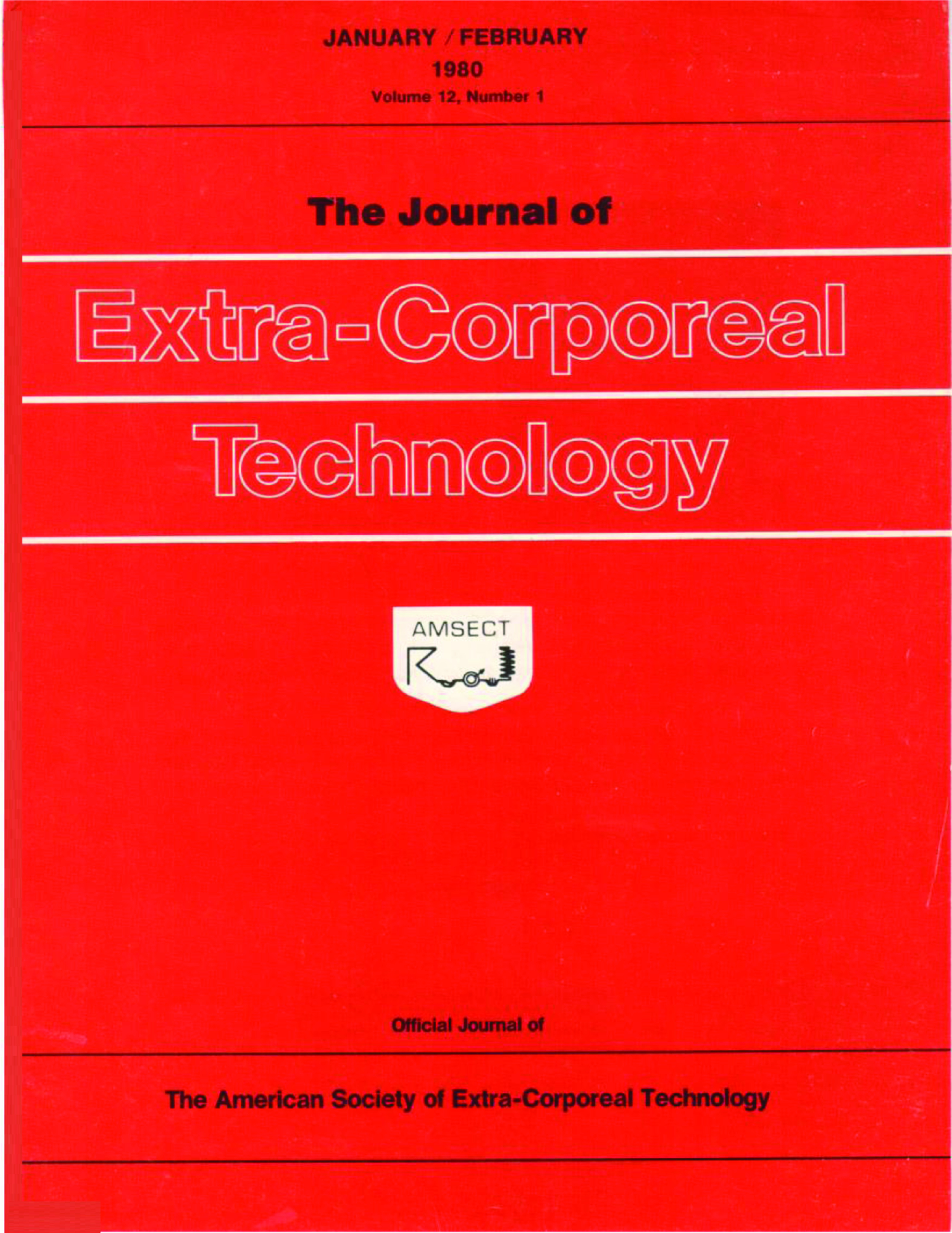
*The Journal of Extra-Corporeal Technology* cover. Jan–Feb 1980. Volume 12, Issue 1.

**Figure 6:**
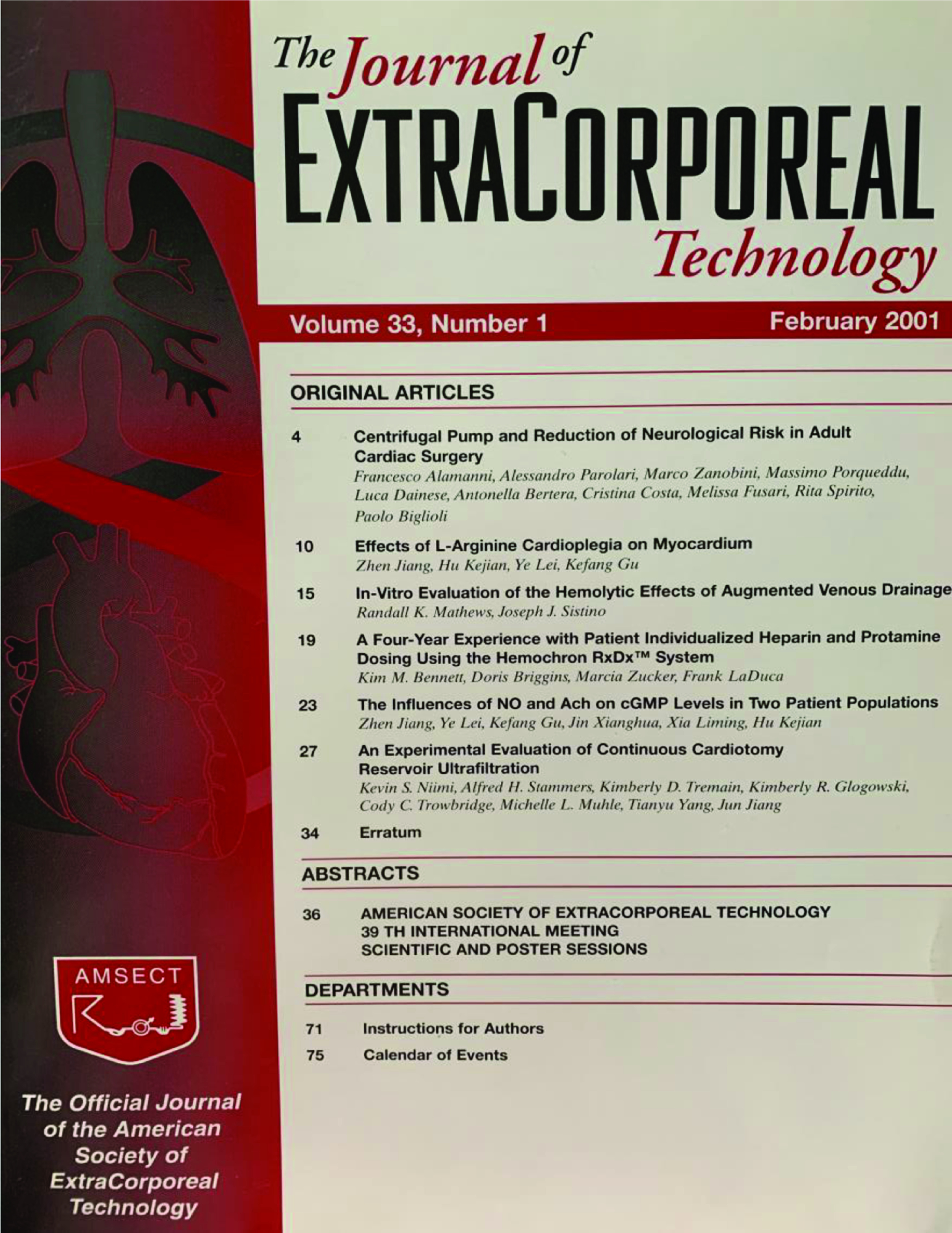
*The Journal of ExtraCorporeal Technology* cover. February 2021. Volume 33, Issue 1. Note the dropping of the hyphen between “Extra” and “Corporeal”.

**Figure 7:**
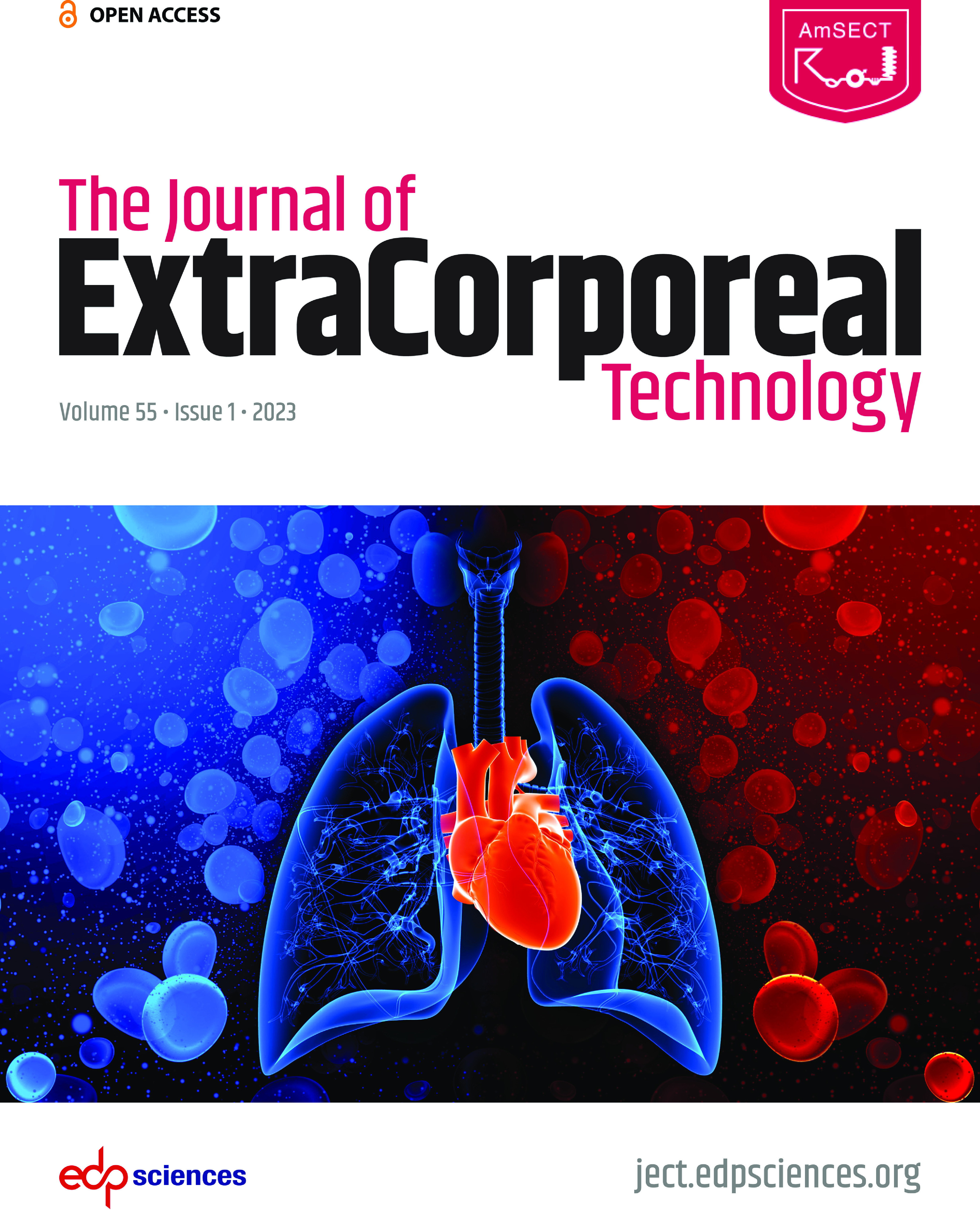
*The Journal of Extra-Corporeal Technology* cover. March 2023. Volume 55, Issue 1.

This format was in continuous use up to the Spring of 1976 when the cover changed to that depicted in Figure 4. While this is shown as a later issue from 1976, the style was the same with the *Journal’s* name all in lower case letters. Then in the first issue of Volume 12, published in early 1980, the cover once again was changed to a solid red background and the AmSECT logo changed from the familiar red with to white with black lettering (Figure 5). Then a rather dramatic change occurred in Volume 33, issue 1 with the addition of a graphic depicting the heart and lungs and an image of an extracorporeal circuit. Here the table of contents was shown on the cover page and the hyphen separating “Extra” and “Corporeal” was eliminated (Figure 6). This cover remained in existence for 22 years and with this issue has been updated maintaining the heart and lungs as well as arterial and venous blood (Figure 7). One constant on the cover has always been the familiar AmSECT logo that first appeared on the cover of a meeting that took place in Montreal in 1967. The emblem was designed by Pierre Morin, Ian Ross, Roger Samson and Jacques Lussier and depicts from left to right a bubble oxygenator with stylized helix, a roller pump, on to the patient shown as a circle with and arrow indicating man in general, then a stylized finger pump with blood flowing from the patient to a coiled kidney [3].

Regardless of the cover graphics, the *Journal* remains the standard bearer for the dissemination of knowledge on technologies associated with extracorporeal blood flow and the editorial staff hope you enjoy both this change and the contents of the publication.


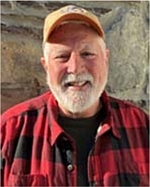


Alfred H. Stammers (MSA, CCP) (Emeritus)

Editor-In-Chief (Emeritus)

Email: al.stammers@specialtycare.net

Phone: (570) 864-8653

## Footnotes

1 It is unknown the exact year when the hyphen separating “Extra-Corporeal” was dropped, but most believe this occurred somewhere prior to 2001.

2 Personal communication with Gary Grist, RN, CCP (Emeritus).
